# A systematic review investigating the relationship between green and blue spaces and depression in older adults via DNA methylation

**DOI:** 10.1093/eep/dvag009

**Published:** 2026-03-14

**Authors:** Jacob Illyuk, Sophie Glover, Shea A Walsh, Anna Jurek-Loughrey, Amy Jayne McKnight, Ruth F Hunter

**Affiliations:** Centre for Public Health, Queen’s University Belfast, Institute of Clinical Science, Block B, Royal Victoria Hospital, Belfast, BT12 6BA, United Kingdom; Centre for Public Health, Queen’s University Belfast, Institute of Clinical Science, Block B, Royal Victoria Hospital, Belfast, BT12 6BA, United Kingdom; Centre for Public Health, Queen’s University Belfast, Institute of Clinical Science, Block B, Royal Victoria Hospital, Belfast, BT12 6BA, United Kingdom; Centre for Public Health, Queen’s University Belfast, Institute of Clinical Science, Block B, Royal Victoria Hospital, Belfast, BT12 6BA, United Kingdom; Centre for Public Health, Queen’s University Belfast, Institute of Clinical Science, Block B, Royal Victoria Hospital, Belfast, BT12 6BA, United Kingdom; Centre for Public Health, Queen’s University Belfast, Institute of Clinical Science, Block B, Royal Victoria Hospital, Belfast, BT12 6BA, United Kingdom

**Keywords:** DNA methylation, green and blue space, green space, blue space, epigenetics, depression, older adults, mental health

## Abstract

Depression in older adults has been associated with negative health outcomes, such as dementia. Previous research has demonstrated that green and blue spaces, defined as areas of vegetation or bodies of water respectively, are beneficial to mental health, although the biological mechanisms are poorly understood. One of the mechanisms proposed is DNA methylation (DNAm). DNAm is an epigenetic process that alters gene expression. Changes in methylation have been observed in those with depression, and associated with green space exposure, while blue spaces have been shown to reduce the risk of depression. Using a mechanistic review approach, we investigated the relationships of green space and depression with DNAm with the aim of identifying potential overlapping mechanisms. In the environmental search, keywords such as ‘green space’ and ‘DNAm’ were combined. In the mental health search, keywords such as ‘DNAm’ and ‘depression’ were combined. From a total of 45 695 papers returned, four studies on green space, and five studies on depression met the eligibility criteria for this review. All included studies reported significant or suggestively significant methylation sites. No overlapping CpG sites were identified when comparing methylation changes found in response to green space and depression. Changes in the *RGS12* gene were associated with both depression and green space exposure. DNAm is a biological mechanism that may contribute to the impact of exposure to green space; further research is warranted to better understand DNAm as a mechanistic pathway between green space and depression.

## Introduction

Depression is one of the most common mental health conditions globally [1]. In 2019, 280 million people worldwide were living with depression [2]. The global rate of depression in older adults is estimated to be 14% [3]. Older adults with neurodegenerative disorders, such as Alzheimer’s Disease (ad) are at an increased risk of developing depression [4]. Evidence suggests that midlife depression is associated with higher rates of cognitive decline and dementia later in life [5]. However, there is also evidence to suggest that lifestyle factors, such as diet and exercise [6], and the surrounding environment modify depression risk [7]. Reducing depression risk not only improves public mental health but may also serve as a modifiable factor for preventing and improving outcomes for neurodegenerative disorders.

Previous research has shown that green and blue spaces (GBS) have a positive impact on mental health [8–13], and have been shown to be especially beneficial for depression [. Green spaces are typically defined as areas of vegetation or plant matter, such as trees, parks, and gardens, while blue spaces are defined as bodies of water, such as seas and lakes. An association between accessing green spaces and mental health outcomes has been established in previous research [15–17]. For example, White *et al*.’s [11] study included 16 307 participants from the BlueHealth study, which included data from 18 European and non-European countries. The study found that accessing green spaces reduced the likelihood of medication for depression being prescribed (OR = 0.99, *P* < .05) [9]. Alcock *et al*. [8] observed that people moving to a neighbourhood with higher residential greenness had improved mental wellbeing, which was sustained over time.

However, the evidence on the long-term impact of green space exposure remains inconclusive. Geneshka *et al*.’s [17] review did not find a statistically significant improvement in mental or physical health associated with GBS. While six of the studies included in this review did not demonstrate a reduction in depression risk associated with green space exposure, two studies found a statistically significant reduction in depression risk [18]. Difficulty in measuring GBS exposure longitudinally was cited by the authors as a potential reason for these findings. Banay *et al*. [7] found a 13% decrease in depression risk associated with increased green space in a 250 m buffer zone (95% CI: 0.78, 0.98), and a 10% decrease in depression risk within a 1000 m buffer zone (95% CI: 0.80, 1.02). Picavet *et al*. [9] observed that higher levels of green within a kilometre of participants residence was associated with a lower depression score (β value = −0.27, 95% CL = − 0.42; − 0.11) and lower rates of depression (OR = 0.86, 98% CL = 0.79; 0.93).

The impact of blue spaces on mental health has been less investigated than green spaces. However, research indicates that blue spaces also have a positive effect on depression [14,15]. A systematic review by Georgiou *et al*. [15] explored the impact of blue space exposure on human health through the following mediators: physical activity, restoration, social interaction, and environmental factors, such as improved air quality and reduced heat stress, and found that blue spaces were associated with reduced prevalence of major depressive disorder, a decreased history of depression, and decreased negative feelings. However, the review also notes that none of the included studies discussed which biological pathways might underlie these changes.

Regulation of nervous system activity has been proposed as a biological pathway through which GBS’s decrease the risk of depression [16,20]. Studies have suggested that exposure to green spaces may reduce sympathetic nervous system activity and increase parasympathetic nervous system activity [19–21], reducing feelings of stress. Stress, especially prolonged stress, is a known risk factor for developing a mental health condition [22–24]. The hypothalamic–pituitary–adrenal (HPA) axis is integral in the stress response [25], and dysregulation in the HPA axis has been implicated in the pathogenesis of depression [26,27]. Decreased allostatic load has been associated with GBS [28]. Gou *et al*. [27] conducted a study with 333 017 participants from the UKB cohort. A positive correlation was found between increased allostatic load and depression risk (hazard ratio = 1.389, *P* = 8.38 × 10−27), which was especially prominent in women.

Despite evidence of the observed positive effects of GBS on mental health, the biological mechanisms remain under researched [16,28–30]. Understanding biological mechanisms underlying disease can increase resilience and aid prevention efforts [31]. Through understanding the biological mechanisms, public health interventions can be developed aimed at those who are at risk of developing a condition. This may be of particular interest in depression due to the interaction of biological and nonbiological factors.

Changes in gene expression underlie several theories currently being explored in depression research [32–36]. DNA methylation (DNAm) is a reversible epigenetic change that occurs when a methyl group is added to a CpG site on the DNA strand, altering gene expression [37]. Changes in methylation patterns have been observed in those with mental health conditions, such as depression [36,38], and in response to green space exposure [39]. In this review, we investigated DNAm as a possible mechanism to explain the effects of GBS exposure on mental health due to the ability of the surrounding environment to influence methylation.

When conducting our preliminary searches, we found no studies exploring the role of DNAm in mediating the relationship between GBS and depression. The interdisciplinary nature of conducting such research presents challenges, such as incompatible study designs, variations in measures used, and a lack of collaboration across fields [40]. However, studies exploring specific parts of the pathway, such as GBS and DNAm [41], and mental health and DNAm [41,42–46] were found when conducting preliminary searches. Through conducting a mechanistic review [47], a type of review which integrates two systematic review searches to identify biological pathways which may connect an exposure and outcome, we aimed to explore the evidence for the contribution of DNAm to the biological pathway between GBS and depression. This type of review gathers evidence on potentially plausible mechanisms common to both an exposure and outcome to promote further research of these mechanisms and test the strength of the association found.

Guidelines for conducting mechanistic reviews were developed by the World Cancer Research Fund and the University of Bristol [47]. Two searches are conducted, and the data from both searches is then synthesised.

This review aims to use a mechanistic review approach to investigate the relationship between GBS exposure, depression, and DNAm. Objectives include to:

1. Conduct a systematic review examining the relationship between GBS exposure and DNAm.
2. Conduct a systematic review examining the relationship between depression and DNAm.
3. Synthesise the data from both reviews using a narrative synthesis.

## Results

### Environmental focus

**Figure 1:**
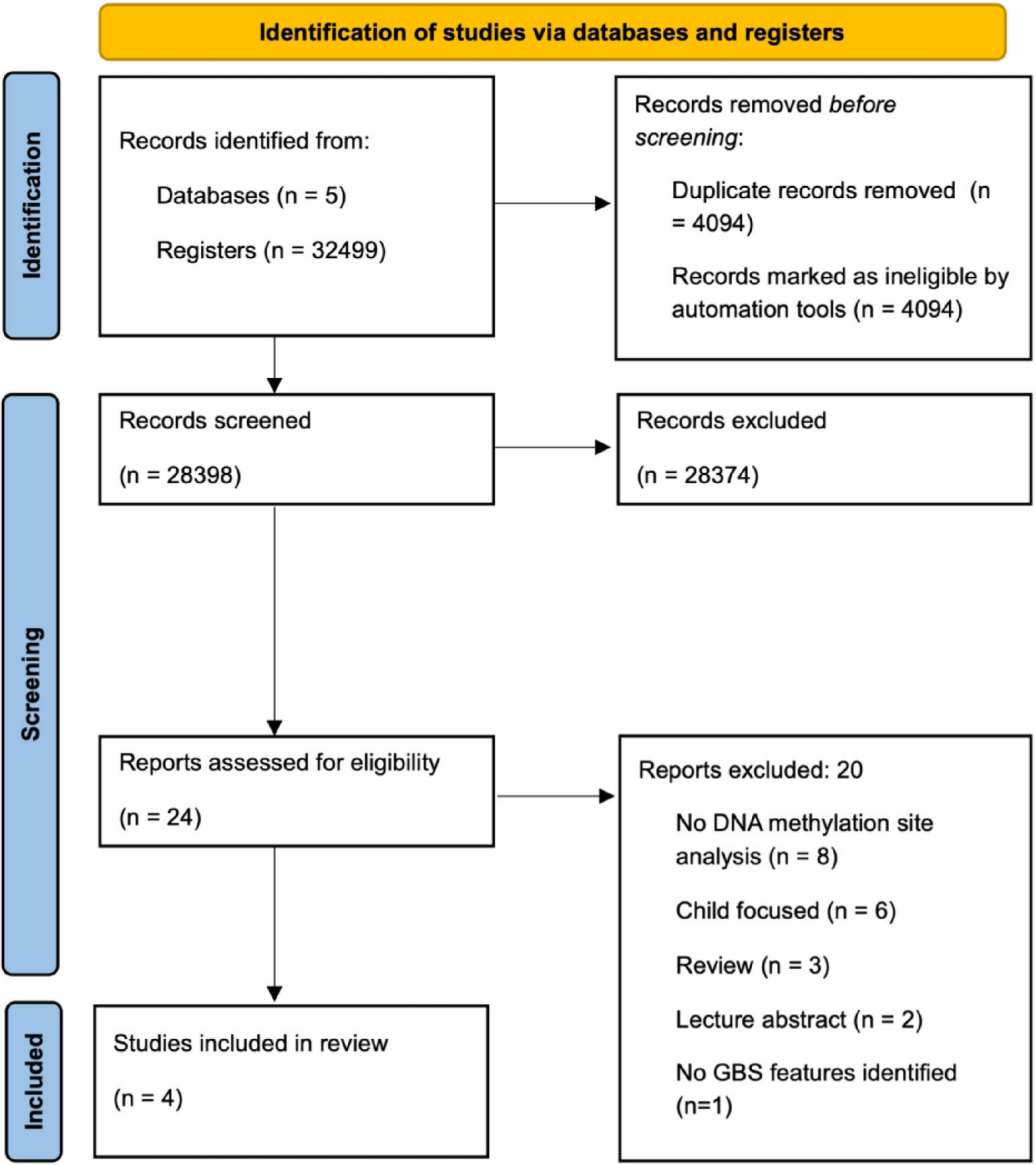
PRISMA diagram detailing the screening process for the environmentally focused search. Template source: PRISMA 2020.

Studies identified from our search were imported into Covidence (*n* = 32 499). Following removal of duplicates (*n* = 4 101), remaining studies (*n* = 28 398) underwent title and abstract screening. The agreement rate between reviewers was very high (99.9%). At the full text screening stage, 24 studies were screened by two independent reviewers. In line with our criteria, four studies were eligible for inclusion in this review. The main reasons for exclusion during full text screening were studies in children (*n* = 6) and studies did not include investigation of DNAm sites (*n* = 8). The full screening process is shown in Fig. 1. Despite our search strategy encompassing both GBS, no studies on blue space met our eligibility criteria. The green space measures used within these studies were normalised difference vegetation index (NDVI), ‘total green’ and enhance vegetation index (EVI) in buffer zones from participants’ residence. Vos *et al*. [48] defined total green as the sum of greenery higher than 3 m plus the sum of greenery lower than 3 m. Results for the risk of bias assessments for these studies are available in Supplementary file 3: risk of bias.

### Green space modified DNAm

#### Study characteristics

Three studies included in this review explored the impact of surrounding residential green spaces on DNAm. Residential green space measures green space surrounding participants’ residential address. Xu *et al*. [39] and Jeong *et al*. [47] conducted EWAS’s, while Vos *et al*. [48] tested methylation in the *NR3C1* and *IGF2/H19* genes. All studies were observational longitudinal cohort studies. Xu *et al*. [39] used a cohort of monozygotic or dizygotic twins, and their sisters, originally recruited for a breast cancer study. Jeong *et al*.’s [47] participants were part of the Study on Air Pollution And Lung Disease In Adults (SAPALDIA). Vos *et al*. [48] investigated the impact of green spaces during pregnancy and maternal stress on DNAm in adult offspring. General study characteristics are described in Table 1.

**Table 1:** Study characteristics of studies investigating the direct impact of residential greenness on DNAm.

Author	Number of participants	Age in years (median)	Percentage female (%)	Ethnicity	Country (income status)	Type of green space	Green space measure
Xu *et al*. [39]	479	55.7	100	Mainly European ancestry	Australia (high income)	Residential	EVI and NDVI
Jeong *et al*. [47]	982	50.2 at 1st timepoint and 58.2 at 2nd timepoint	56.3	8 centres in Switzerland included, with varying demographics	Switzerland (high income)	Residential	NDVI
Vos *et al*. [48]	88	27.8 at saliva collection and 29.3 at blood collection	51.8	100% Caucasian	Netherlands (high income)	Residential	Total green

EVI—enhanced vegetation index.

NDVI—normalised difference vegetation index.

Total green—a value calculated from land map data. Total green = sum of greenery lower than 3 m + sum of greenery higher than 3 m.

#### Evidence synthesis—DNAm sites

Xu *et al*. [39] conducted an EWAS. In Xu *et al*.’s [39] study, after FDR correction only one CpG site, cg04720477, was significantly associated with EVI within 300 m of participants’ residence (*q* value = 0.026028316). This CpG site lies on chromosome 17 and is associated with the promoter region of the *CNP* gene. Jeong *et al*. [47] also conducted an EWAS but found no significant associations after Benjamini–Hochberg correction. Both Xu *et al*. [39] and Jeong *et al*. [47] found additional CpG sites that were significantly correlated with green space exposure prior to multiple hypothesis testing correction and were deemed ‘suggestively significant’. Vos *et al*. [48] assessed methylation in the *IGF2/H19* and *NR3C1* genes. Within a 1000 m buffer zone from participants residence, methylation was altered in the *IGF2/H19* gene at CpG95 (*P* = .04) and CpG101 (*P* = .05), located on chromosome 11. This change was only observed in saliva.

### Green spaces and reduced epigenetic mortality risk

#### Study characteristics

One study focused on the mitigatory effect green space features have on mortality risk. Ward-Caviness *et al*. [49] conducted a longitudinal cohort study in the USA (Detroit Neighbourhood Health Study). The cohort was predominantly female (61.8%), with a mean age of 53.3 years, and most participants were from a ‘black or African American’ background (87.9%). Methylation analysis included 157 participants. Although the USA is a high-income country, this study included deprived neighbourhoods in Detroit. Measures of neighbourhood deprivation, such as the condition of streets in the neighbourhood, were assessed. Socio-economic status was assessed using employment level (29.3% employed) and education level obtained (17.8% graduated college or had a postgraduate degree).

#### Evidence synthesis—DNAm sites

DNA was extracted from whole blood. Methylation was assessed in ten CpG sites. These sites comprise an epigenetic mortality risk score (eMRS), developed by Zhang *et al*. [50]. Neighbourhood deprivation increased eMRS via changes in DNAm. However, the presence of large trees and gardens reduced the eMRS. Changes in DNAm in six CpG’s associated with increased mortality risk were not statistically significant in the presence of large trees and gardens (*P* > .05) but were significant in the overall analysis (*P* < .05). A summary of the features associated with reduced mortality risk is presented in Table 2.

**Table 2:** Neighbourhood features associated with a reduced eMRS across six CpG’s in the Detroit Neighbourhood Health Study. If *P* ≤ .05, a significant increase in mortality was reported in the study. In the presence of neighbourhood green space features, such as large trees and gardens, methylation levels at these six CpG sites no longer indicated an increased mortality risk in a statistically significant manner.

CpG Site	Mortality risk *P*-value	Neighbourhood feature	Mortality risk *P*-value
cg01612140	4.0 × 10−4	Large trees	.69
cg08362785	4.5 × 10−4	Gardens	.17
		Large trees	.37
cg10321156	.02	Gardens	.65
		Large trees	.86
cg23665802	.001	Gardens	.14
		Large trees	.17
cg24704287	.002	Gardens	.15
		Large trees	.38
cg25983901	.02	No gardens	.21
		Gardens	.32
		Large trees	.67

#### Depression focus

**Figure 2:**
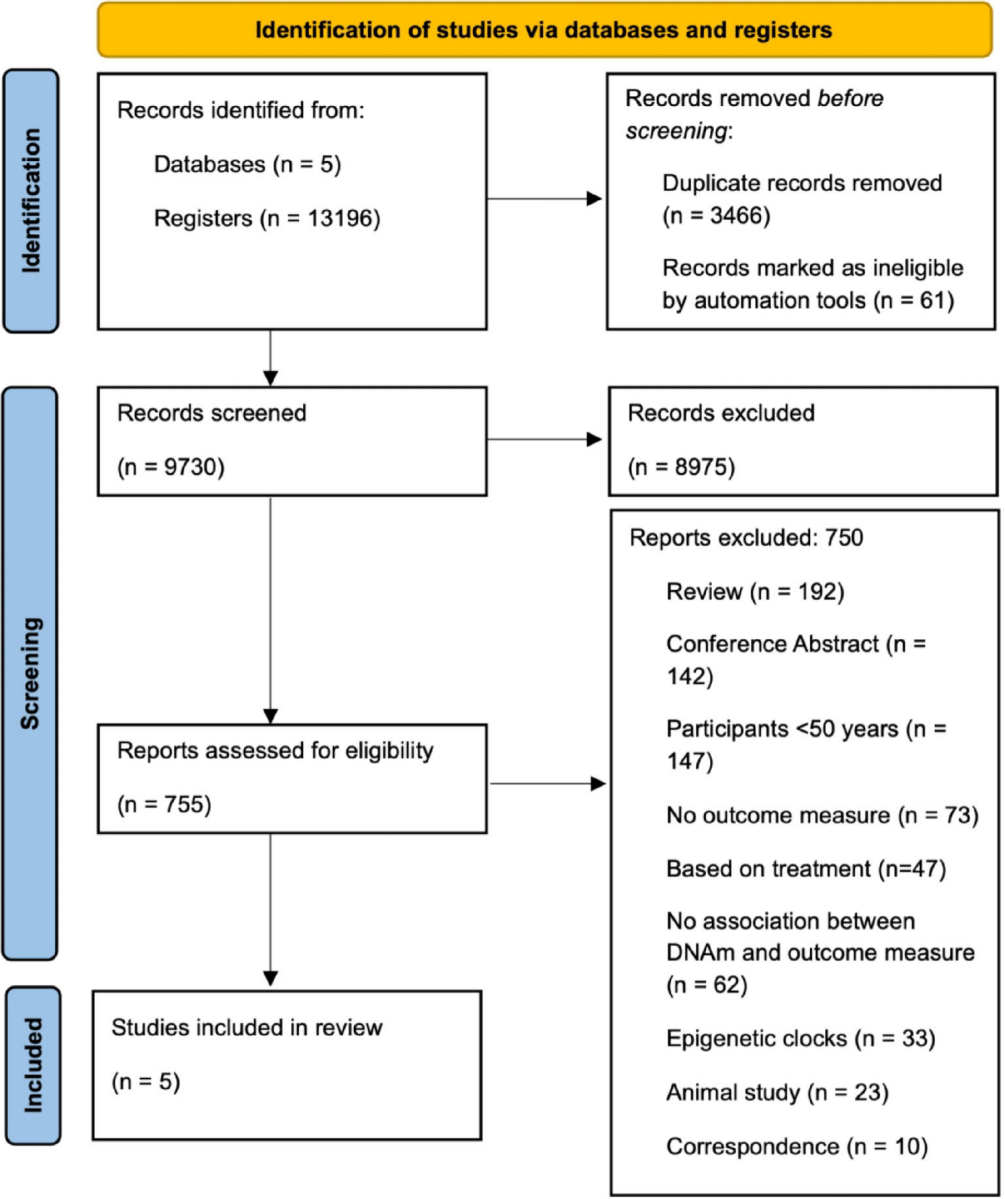
PRISMA diagram detailing the screening process for the depression focused search. Template source: PRISMA 2020.

Our search yielded 13 196 studies. Following removal of duplicates, 9730 studies underwent title and abstract screening. Full text screening included 755 studies. According to our criteria, five studies were deemed relevant for inclusion in this review. Studies were screened by two independent reviewers, and the agreement rate was above 95% at all stages. The main reasons for exclusion at the full text screening stage were review studies (*n* = 189) and studies in those under 50 years old (*n* = 147). The full screening process is shown in Fig. 2. Results for the risk of bias assessments for these studies are available in Supplementary file 3: risk of bias.

#### Study characteristics

Studies included in this review conducted analysis in older, community dwelling adults (aged over 50 years). All studies included in this review are cohort studies and examined the impact of depression on DNAm. All studies, except for Liao *et al*.’s [51] study were conducted in high income countries. Liao *et al*.’s [51] study was conducted in China, an upper middle-income country. Two studies (Kang *et al*. [52] and Kang *et al*. [53]) administered the Geriatric Mental State B3 psychometric test to participants, that was then converted into an AGECAT score via a computer algorithm. Depression was defined in these studies as an AGECAT score ≥ 3. Both studies used the same longitudinal cohort. Januar *et al*. [109] used the Centre for Epidemiologic Studies Depression Scale (CES-D) to assess depression, with a score ≥ 16 allocating participants to the depression group. Liao *et al*. [51] used the Patient Health Questionnaire 9 (PHQ-9) to assess depression symptoms, without a cutoff point to denote depression. Starnawska *et al*. [54] used the Cambridge Mental Disorders in the Elderly (CAMDEX) to assess depression. General information about the studies is available in the Table 3.

**Table 3:** Study characteristics of studies exploring the impact of depression on DNAm. Starnawska *et al*.’s [54] study used two cohorts from a longitudinal twin study: The Middle Age Danish Twins Study (MADT) and The Longitudinal Study of Aging Danish Twins (LSADT). Both studies conducted by Kang *et al*. [52] used the same cohort. Participants were invited to the follow up study only if they did not report depression at baseline.

Author	Subgroup	Number of participants	Age in years (mean)	Percentage female (%)	Ethnicity	Country (income status)	Depression measure
Januar *et al*. [109]	Not depressed	773	71.4	55	Not stated	France (high)	CES-D
	Depressed	251	72	77			
Liao *et al*. [51]	Not depressed	18	66.58	66.67	Not stated	China (upper-middle)	PHQ-9
	Depressed		70.5	33.33			
Starnawska *et al*. [54]	MADT	486	65.9	45.7	Not stated	Denmark (high)	CAMDEX
	LSADT	238	77.95	65.5			
Kang *et al*. [52]	Baseline	732	72.8	59	Not stated	South Korea (high)	AGECAT (GMSB3 based)
	Follow-up	521	72.5	55.1			
Kang *et al*. [53]	Baseline	732	72.8	59	Not stated	South Korea (high)	AGECAT (GMSB3 based)
	Follow-up	521	72.5	55.1			

#### DNAm

Starnawska *et al*.’s [54] study and both studies conducted by Kang *et al*. [53] used blood samples to obtain methylation data. Januar *et al*. [109] used buccal tissue and Liao *et al*. [51] used saliva. Only one study examined methylation across the whole epigenome (Starnawska *et al*. [54]), while other studies examined methylation changes in specific genes: *BDNF* (*n* = 3) and *NR3C1* (*n* = 1). All studies established a relationship between depression and changes in the methylome.

### Evidence synthesis—DNAm sites

#### BDNF

Three studies explored the impact of depression on *BDNF* methylation (Liao *et al*. [51], Januar *et al*. [109], and Kang *et al*. [53]). All three studies found statistically significant associations between depression and methylation in the *BDNF* gene. Liao *et al*. [51] found significant changes in loci 1–9 associated with depression and in loci 1–5 and 7–9 when comparing methylation in depressed and nondepressed participants, as shown in Table 4.

**Table 4:** Methylation sites identified in Liao *et al*.’s [51] study, which were significantly associated with depression.

Lead author	Methylation site	Methylation and depression *P*-value	Depressed vs nondepressed *P*-value
Liao	Locus 1	.006	.031
	Locus 2	.002	.037
	Locus 3	<.001	.015
	Locus 4	.006	.039
	Locus 5	<.001	.004
	Locus 6	.008	.055
	Locus 7	<.001	.002
	Locus 8	<.001	.003
	Locus 9	.012	.049

After adjusting for age, sex, antidepressant use, and functional impairment, Januar *et al*. [] identified significantly increased methylation at CpG 3.4.5 promoter 1 (*P* = .001). Januar *et al*. [] also found significant associations between CpG 3.4.5 promoter 1 methylation participants with a minor allele of rs6265 (*P* = .0001) and rs7103411 (*P* = .0002) and those with homozygous major alleles of rs908867 (*P* = .006). Follow-up analysis also revealed increased methylation at CpG 3.4.5 promoter 1 (*P* = .0019) and CpG 3 promoter IV (*P* = .0061), and also at CpG 1 promoter 1 (*P* = .016).

Kang *et al*.’s [52] multivariate analysis revealed higher methylation at CpG9 [OR 1.02, CI (1.01–1.02)] and average CpG methylation [OR 1.06, CI (1.03–1.09)] in participants with prevalent depression at baseline. At the 2-year follow-up, those that had no depression at baseline but were depressed at the follow-up also had increased methylation at CpG9 [OR 1.02, CI (1.01–1.03)] and increased average methylation [OR 1.06, CI (1.02–1.09)]. Kang *et al*. [53] also found that depression symptom severity correlated with methylation at CpG9 (*P* = .005) at baseline and average CpG methylation at baseline (*P* = .003) and follow-up (*P* ≤ .001).

#### NR3C1

Kang *et al*. [52] assessed methylation in the exon 1F region of the *NR3C1* gene. A statistically significant increase in methylation was observed at CpG2 (*P* = <.001) and average methylation percentage (*P* = .005) was associated with depression at baseline and was significant after Bonferroni Correction in unadjusted models. In multivariate analysis at baseline, CpG2 (OR = 1.08), CpG3 (OR = 1.07), and average CpG (OR = 1.09) were significant after Bonferroni Correction. At the 2 year follow up, CpG2 was significantly correlated with depression (OR = 1.08).

#### EWAS

Starnawska *et al*. [54] pooled two twin cohorts for their study. In their paired twin model, cg05777061, located on the *KLK8* gene, was associated with depressive symptoms (*P* = 4.70E-07) following FDR correction. In the unpaired twin model, cg0554948, located on the *DAZAP2* gene, was associated with depressive symptoms (*P* = 3.13E-08) following FDR correction. Several suggestively significant CpG sites (0.05 < FDR < 0.10) were also found across multiple genes.

### Mechanistic evidence synthesis

#### Green space and depression

No CpG sites identified in the environmental studies matched any of the CpG sites identified in the depression studies.

Interestingly, changes in the *RGS12* gene were associated with both green space and depression. Jeong *et al*. [47] found an increase in methylation at the cg08817983 site in response to greenery within 30 m of the participants residence. Starnawska *et al*. [54] observed an increase in methylation at the cg01919885 site in depressed participants. However, these sites were only suggestively significant.

#### Risk of bias

The full results of the risk of bias screening can be found in Supplementary file 3: risk of bias. Three studies were assessed using BIOCROSS and six studies were assessed using the Joanna Briggs Institute (JBI) cohort study assessment tool. The mean BIOCROSS score was 12. In the BIOCROSS assessment, all studies scored highly in the Statistical Analysis and Interpretation and Evaluation of results sections. All studies performed poorly in the Study Limitations category of the assessment. In the JBI cohort study assessment, studies performed well in the Population, Exposures, Exposure Measures, and Reliable Outcome Measure domains but were largely unable to ensure participants were free of the outcome prior to exposure. In the BIOCROSS assessment, studies from the depression focused search performed better than studies from the environmentally focused search. Two depression focused studies scored 13 points, while Ward-Caviness *et al*.’s [49] environmental study scored 10 points. Scores differed the most in the Biomarker Modelling section, with depression studies scoring higher. Three studies were assessed using the JBI cohort assessment tool from each half of this review, with depression focused studies overall performing similarly to environmental studies.

## Discussion

**Figure 3:**
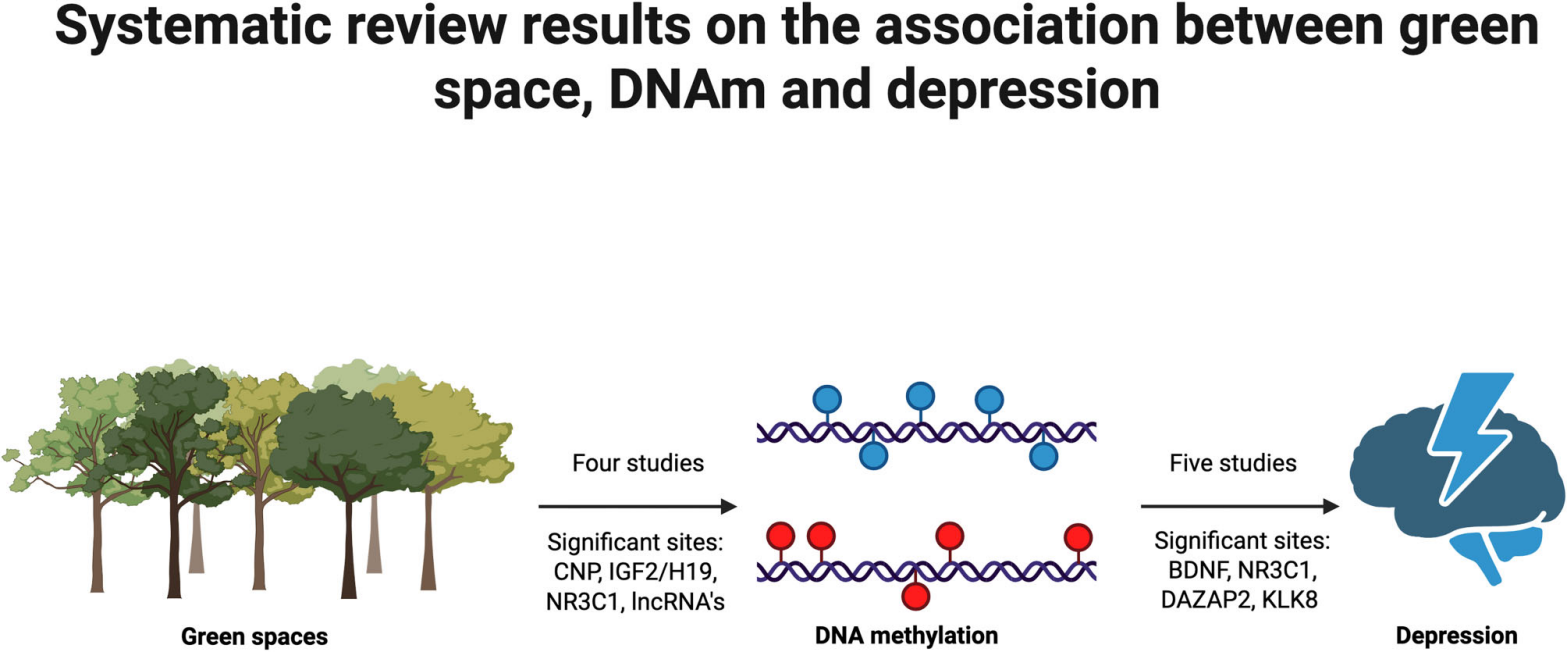
A summary of the evidence identified from the environmental search and mental health focused search to support the relationship between green spaces and DNAm and depression and DNAm. The environmental focused search included four studies, which met our inclusion criteria, while the depression focused search included five. Significant sites from each section of the review are also stated. Significant sites were identified based on the significance level established by the researchers in the original study. Created in BioRender. Illyuk, J. (2026) https://BioRender.com/c4t46j0.

This review explored the role of DNAm in influencing the relationship between green spaces and depressive symptoms and found several methylation sites associated with green spaces, and with depression independently. A diagram summarising our findings is shown below (Fig. 3).

Differential methylation in *RGS12* was also associated with both green space and depression to a suggestively significant level. However, this review also highlighted significant gaps in the research in this field. Most significantly, no study met our eligibility criteria exploring GBS, depression and DNAm in the same cohort. This has inhibited our ability to test mechanistic pathways and may have influenced the methylation sites identified due to differences in genotype across cohorts. Additionally, the distinct cohorts used in each section of this review impair our ability to establish causal relationships, and therefore, infer mechanistic pathways. It is difficult to draw correlations between depressive symptoms and green space exposure across cohorts, which impairs the ability to explore potential mechanistic pathways. The lack of blue space research is another major gap in this field. We were unable to find any studies exploring methylation changes associated with blue spaces, despite evidence of their impact on health [14,15]. Based on the results of this review, we have identified several recommendations to improve knowledge in this field.

### Gene expression

Although this review failed to identify methylation in overlapping CpG sites associated with the environment and depression, methylation changes in the *RGS12* gene associated with depression and green space were identified. We present a hypothesis through which differential *RGS12* methylation may potentially influence depression outcomes in relation to green space exposure. *RGS12* is expressed predominantly in the digestive tract and brain [60]. In the brain, the highest expression of *RGS12* is in neuronal cells [60]. In a murine model, rats with altered *RGS12*, which were exposed to chronic stress had an increased risk of developing depression [33]. In another murine model, knockout *RGS12* rats had decreased levels of serotonin [34], a neurotransmitter implicated in the pathogenesis of depression [56–59]. Interestingly, increased methylation associated with depression was found at a CGIa site [50], which has been implicated to result in gene silencing [63]. This is consistent with the results of the murine studies, in which decreased *RGS12* expression increased the risk of depression. Although the methylation change found in Starnawska *et al*.’s [57] study likely leads to silencing of *RGS12* [63], the effect of the change in methylation identified in Jeong *et al*.’s [47] study associated with green space exposure remains less clear. The identified CpG site is located in the gene body [64], and research suggests a positive correlation between gene body methylation and gene expression may exist [61,65].

One of the potential mechanisms through which *RGS12* methylation may contribute to depression is through protecting serotonin receptors from overstimulation. In humans, 13 out of 14 serotonin receptors are G Protein Coupled Receptors (GPCR’s) [62,66]. GPCR’s are membrane bound receptors [67], which respond to external cell signals, triggering a downstream cascade inside the cell [67], contributing to functions such as mood regulation [68]. *RGS12* is a Regulator of G Protein Signalling (RGS) [69], which regulates GPCR function through increasing GTPase activity [69]. This increases the speed at which the GPCR deactivates [69], protecting it from overstimulation [70]. If a GPCR becomes overstimulated, desensitisation commonly occurs [70,71], resulting in a decrease or loss of function [70,71]. Loss of serotonin receptor function, such as through desensitisation caused by *RGS12* methylation likely resulting in gene silencing as found by Starnawska *et al*. [57], may be a mechanism contributing to the pathophysiology of depression in older adults which warrants further exploration.

Another mechanism through which *RGS12* has been shown to alter serotonin is through modulating the amount of serotonin produced [34]. In a knockout *RGS12* rat model, *RGS12* silencing was associated with decreased serotonin production [34]. Decreased levels of serotonin reduce serotonin receptor activation [72], which reduces downstream signalling and increases the risk of depression [72]. Some studies have found evidence implicating low serotonin levels in depression [72]. Increasing available serotonin through inhibiting serotonin reuptake is the mechanism targeted by SSRI drugs [73], the most common pharmacological treatment for depression [73–76]. Although a relationship between serotonin levels and depression has been established in some studies [72], a review by Moncrieff *et al*. [73] found that the evidence of serotonin concentration being correlated with depression was inconsistent, suggesting other biological processes may be involved [78].

**Figure 4:**
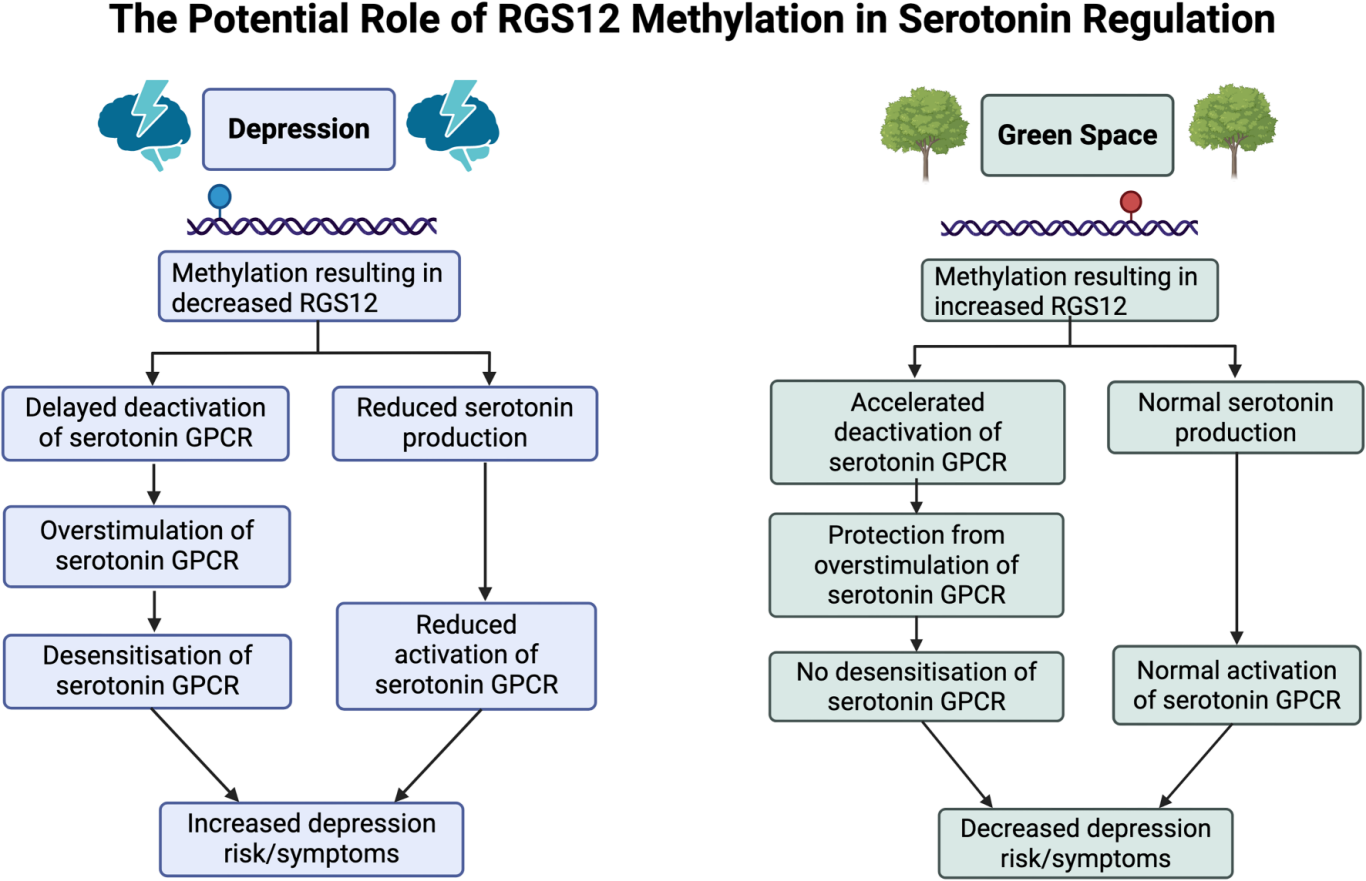
A diagram detailing the potential biological changes in *RGS12* associated with depression and green space. Methylation resulting in gene silencing in *RGS12* may reduce serotonin production and increase the risk of overstimulation and desensitisation of serotonin receptors, which has been associated in research with increased risk of depression and depression symptoms. Increased methylation in *RGS12* has been associated with green space exposure and may help maintain serotonin production and protect the serotonin receptor from overstimulation. Created in BioRender. Hill, C. (2026) https://BioRender.com/47n5q61.

If methylation at the CpG site identified by Jeong *et al*. [47] associated with green space exposure results in upregulated *RGS12* expression, this could imply that green spaces may reduce symptoms of depression and depression risk through supporting normal serotonin function, as shown in Fig. 4. We propose two hypothetical mechanistic pathways through which green spaces may influence depression: increasing serotonin availability, and modulating serotonin receptor function.

### Depression in older adults

Changes in *IGF2* expression and serum concentration have been found in the literature to be associated with depressive symptoms [79,80]. However, the studies included in this review did not find methylation changes in *IGF2* in depressed older adults. This may be due to depression studies in older adults focusing on methylation in specific genes, rather than across the epigenome. Differential methylation in *IGF2* may be of particular exploratory interest in older adults. Age is a significant risk factor for developing ad [81], and patients with depressive symptoms suffer from worse cognitive and overall outcomes [82]. Luo *et al*.’s [74] study revealed altered *IGF2* methylation in patients with ad and major depressive disorder, but patients with ad alone did not show altered *IGF2* methylation [79]. Further EWAS’s in older adults with depressive symptoms may be beneficial to identify epigenetic changes associated with depression in this demographic.

Although schizophrenia is a risk factor for depression across the lifespan [84], older adults with schizophrenia have been found to be at a particularly elevated risk of developing depression [85]. In schizophrenic patients, depression may be persistent and remain or develop after a psychotic episode has passed [85,77]. Schizophrenia is usually a chronic condition, resulting in this demographic having a permanently increased risk of depression [85,86]. Similar to depression, schizophrenia is thought to be caused by the interaction of genetic and environmental factors [87]. Green spaces have been shown to be beneficial for schizophrenia [88–90], and there has been interest in developing green space-based interventions for those with schizophrenia [83]. Green spaces reduce the risk of developing schizophrenia [91,92], reduce psychotic symptoms [93] , and decrease hospitalisation [94] in schizophrenic patients. Crucially, multiple studies have found that accessing green spaces also reduces depressive symptoms in those with schizophrenia [95,96].

Polymorphisms in the *CNP* gene have been identified as potential genetic features associated with the development of schizophrenia [97], although some studies have failed to find significant associations between *CNP* polymorphisms and schizophrenia [98]. In the central nervous system, *CNP* regulates oligodendrocytes and myelin formation [99,100]. There is also evidence to suggest that myelin and oligodendrocyte dysregulation is associated with schizophrenia [101,102]. Xu *et al*.’s [39] study identified a methylation change in the *CNP* gene associated with green space, which remained significant after FDR correction [49]. Xu *et al*.’s [39] study included only female participants and was the only study to establish a relationship between green space and *CNP* methylation. It may be of particular interest for future research to explore whether a relationship exists between depressive symptom severity, *CNP* methylation and green space exposure in females. If such a relationship exists, *CNP* methylation may be one of the biological processes underpinning the improvement of depressive symptoms correlated with green space exposure in those with schizophrenia.

Schizophrenia, especially chronic and poorly treated schizophrenia, is characterised by cognitive impairment [103] and there is evidence to suggest that those with schizophrenia are at a significantly increased risk for developing a neurodegenerative disorder [104,105]. Older adults with schizophrenia are particularly vulnerable to developing a neurodegenerative condition, as the risk of developing such a condition increases with age [81,46,106]. Further research into the involvement of the *CNP* gene in schizophrenia, and the ways in which green space associated *CNP* methylation influences depression outcomes, may aid in the development of new interventions, improving quality of life and reducing the incidence of long-term negative health outcomes.

### Reducing depression risk

Three studies included in this review identified methylation in the promoter regions of the *BDNF* gene [54,48,56]. Methylation in the promoter region has been associated with gene silencing [107]. Decreased levels of serum *BDNF* have been found in those with depression [108], while physical activity, a proposed mechanism through which GBSs improve depression outcomes [109] has been shown to increase *BDNF* production [110,111]. A conceptual model has been developed around quantifying the release of *BDNF* in response to the built environment [112,115], which may lead to novel intervention strategies for those with depression.

### GBS research

The risk of mobility impairment increases with age [113], and older adults may become less able to access local green spaces, such as parks. Only one study in this review included buffer zone under 500 m in their analysis. The CpG sites identified in response to increasing green space within a 30-m buffer zone from participants residence did not follow the same trend as CpG sites identified in larger buffer zones from participants residence. Including smaller buffer zones in further studies into the epigenetic impact of green space exposure may produce results that are more relevant to older adults.

We did not identify any studies that met our eligibility criteria that investigated exposure to blue spaces, however, evidence suggests that exposure to blue space is also beneficial to mental health [15]. The lack of epigenetic blue space research is a major gap in the field. Research exploring the methylation changes associated with blue spaces are needed and may aid in harnessing their power for disease prevention.

### Recommendations

A summary of recommendations for future studies in this field is outlined in Table 5.

**Table 5:** Recommendations for future research in this field.

Recommendation 1	Further EWAS’s examining the association between methylation and depressive symptoms in older adults should be conducted. This may lead to DNAm changes in other regions of the methylome associated with depression being missed.
Recommendation 2	Additional studies investigating methylation changes associated with green space and depression including a wider range of ethnicities.
Recommendation 3	Further cohort studies exploring DNAm and depression in the same participants with information measured at repeat timepoints are necessary. Having DNA genetic information, comprehensive epigenetic measures and gene expression would facilitate investigation of these molecular biomarkers and allow causal analysis to be conducted using Mendelian Randomisation.
Recommendation 4	Future research on the potential protective benefits of GBS, as demonstrated in this review by Ward-Caviness *et al*. [49], would be a valuable contribution to the field. Studies which include participants with risk factors for developing depression, gather information about the GBS which surround them, collect DNAm data and follow participants longitudinally may provide useful insight into the protective benefits of GBS
Recommendation 5	Studies to identify changes in DNAm related to blue spaces are needed. Although the benefits of blue spaces have been documented, no studies were found in this review exploring the epigenetic changes associated with blue spaces.
Recommendation 6	Future studies should include measures of publicly accessible green spaces, rather than being limited to NDVI/EVI alone as not all green spaces may be publicly accessible.
Recommendation 7	Green space density and features in smaller buffer zones around participants’ residence should be considered to produce results, which may be more relevant to an older population which may struggle with mobility.
Recommendation 8	Standardised, transparent reporting from sample collection, to reporting of results. This was underreported in many studies included in this review and identified as a shortcoming during risk of bias screening. Increased reporting of strategies for participant retention in longitudinal studies was also identified as an underreported area during risk of bias screening.

### Strengths and limitations

This review used a mechanistic review approach to explore the association between GBS, DNAm, and depression. The results of this review provide insight into the role DNAm may play as a potential biological mechanism influencing the positive impact of GBS on depression. Methylation changes were found in this review in the *RGS12* gene associated with both GBS exposure and depression, showing potential involvement of common biological pathways. Further, the methylation changes observed were consistent with murine research on the potential role of *RGS12* in depression—a reduction in *RGS12* expression was shown to increase the risk of depression.

We did not find any studies exploring GBS, DNAm, and depression outcomes in the same cohort, which has limited our ability to test mechanistic pathways or infer causation. However, the findings of this review, such as suggestively significant methylation identified in *RGS12* associated with both green space and depression, demonstrate that this area warrants further research. If concordant methylation changes are identified in the same cohort, a causal relationship can be established using methods such as Mendelian Randomisation.

A major finding of this review is the lack of blue space studies fitting our inclusion criteria, leaving us unable to explore epigenetic changes associated with blue spaces, and how they may interact with depressive symptoms. This highlights a significant gap in the literature and research in this area may improve understanding of the impact blue spaces have repeatedly been shown to have on depressive symptoms in the literature.

The heterogeneity captured in this review limited our ability to draw comparisons between studies. Key features of both sections of this review are summarised in Table 6 below.

**Table 6:** A summary of the study designs, study types, mean age of participants, and tissues used in the studies included in this review.

Characteristic	Environmental search	Depression search
Mean age (years)	45.75	71.94
Tissue type (number of studies)		
Blood	3	3
Saliva/buccal swab	1	2
Study design (number of studies)		
Cross sectional	2	1
Observational cohort	3	3
Study type (number of studies)		
Number of EWAS’s	2	1
Number of specific gene studies	2	4

The difference in the mean age of participants and tissue types used to obtain methylation data in the environmental and depression focused searches (Table 6) is a further limitation of this review. While the mental health search was limited to adults over 50 years old, the green space search was limited to adults over 18 years old as no green space studies were found that excluded those under 50 years old. However, in three out of four green space studies, the mean or median age of participants was over 50 years old. DNAm is influenced by age [114], and is tissue specific [115], so these discrepancies may negatively influence our ability to compare methylation sites identified across studies.

A further limitation of this review is the lack of EWAS’s relevant to the topic (Table 6). We found only two studies exploring depression in adults over 50 years old. Of the green space studies included in this review, two assessed methylation at specific sites in the epigenome. This limits the interpretation of the results of this review, as other methylation sites, which may be involved in the pathogenesis of depression, and may be influenced by green space exposure, could be important.

The depression focused studies in this review consistently accounted for confounding variables such as sex, medication use, and other health conditions. However, none of the studies included ethnicity in their adjusted analysis, and the ethnicity of participants was not reported in any of the depression focused studies. This may be due to all participants being of one ethnicity, but this was not explicitly stated in the methods of any study. The risk of depression may be influenced by ethnicity, as ethnic minorities are at an increased risk of developing depression. Ethnicity should be included in adjusted analysis, or authors should explicitly state that participants are of the same ethnic background.

## Conclusion

In summary, this mechanistic review found an overall decrease in methylation associated with increasing green space. In participants with depression, DNAm increased at the *BDNF* gene [54,48,56], the *NR3C1* gene [55], and in several CpG sites identified in the EWAS [59]. An increase in methylation at sites in the *RGS12* gene were identified in participants with high depression outcome scores and in association with green space exposure [50,59], consistent with research demonstrating the potential role of *RGS12* in depression in rats. However, methylation at in this gene associated with green space and depression was only found to be suggestively significant in this review. A potential cause of this may be differences in the genetic profiles of participants across cohorts [107]. This review also highlighted several gaps in research in this field, most importantly, the lack of research investigating associations between GBS, depression, and DNAm within a single cohort, or DNAm patterns associated with blue spaces. Several recommendations have been made to address these challenges and improve understanding in this field. GBSs have the potential to be used to improve mental health outcomes, however, more research is needed for this to be realised.

## Methods

For this review, two searches were conducted and data from both searches were synthesised following extraction. Prior to commencing searches, the protocol for this review was registered on PROSPERO (CRD42024531130).

### Search strategy

Searches were conducted in Pubmed, Embase, and PsycINFO from inception until December 2024. Additionally, medRxiv and bioRxiv were searched for any relevant grey literature published within the last year. Two searches were conducted:

1. Environmentally focused search: GBS and DNAm search terms
2. Mental health focused search: depression and DNAm search terms

A summary of the search terms used in this review are outlined in Table 7. The environmentally focused search consisted of environmental and DNAm terms, while the mental health focused search consisted of DNAm and depression terms. The full search strategy for this review is available in Supplementary file 1: search strategy.

**Table 7:** A selection of search terms used in this review.

Environmental terms	DNAm terms	Depression terms
Green space	DNAm	Depression
Blue space	Hypermethylation	Major depressive disorder
Park	Hypomethylation	Mental health
River	Epigenetic	Depressive

Inclusion and exclusion criteria are outlined in Table 8 below.

**Table 8:** Search specific inclusion and exclusion criteria used for this review.

Search	Inclusion criteria	Exclusion criteria
Environmentally focused	Studies which identify a green space (defined as any vegetation or plant matter, such as parks or trees), and conduct association analyses with DNAm	Studies which identify, but do not conduct association analysis between green spaces or blue spaces and changes in DNAm
	Studies which identify a blue space (defined as any body of water, such as lakes or river), and conduct association analyses with DNAm	Studies with participants aged under 18 years
Mental health focused	Studies which use a validated outcome measure for depression, such as the Hamilton Depression Rating Scale, and conduct an association analysis of this outcome with DNAm	Studies which did not complete the outcome measure assessment and collect a tissue/blood sample concurrently
		Studies which did not use a validated outcome measure to assess depression
		Studies which did not conduct an association analysis between outcome measure scores and DNAm changes
		Studies including neurodegenerative or neurodevelopmental conditions, such as autism or Alzheimer’s Disease
		Studies primarily investigating DNAm changes in response to treatment (e.g. medication, psychotherapy, etc.)
		Studies with participants aged under 50 years
Both searches		Studies not in the English language
		Posters, reviews, conference abstracts, and theses

The mental health focused search was limited to adults over 50 years old due to association between age, depression, and dementia . Further details on inclusion and exclusion criteria can be found in Supplementary file 2: inclusion criteria.

### Screening

Studies returned from our search were imported into Covidence. Titles and abstracts of all imported studies were screened by JI. A second independent reviewer (SG or SW) screened 10% of all titles and abstracts. All studies selected for full text screening were screened by two independent reviewers (JI and SG or SW).

### Data extraction

Microsoft Excel was used for data extraction. Templates were created for reviewers to use for each search. Data was extracted by two independent reviewers (JI and SG or SW).

### Risk of bias

All risk of bias assessments were conducted by two independent reviewers (JI and SG or SW). Any disagreements were resolved by a third party (RH, AJM, or AJL). BIOCROSS [116] was used to assess potential bias in cross-sectional studies, while the JBI critical appraisal tool for cohort studies was used for longitudinal studies [117]. Studies were not excluded based on the outcome of the risk of bias assessment. The results of the risk of bias assessments can be found in Supplementary file 3: risk of bias.

### Evidence synthesis

Due to study heterogeneity, meta-analysis was not feasible for this review. Therefore, a narrative synthesis was conducted using the DNAm sites identified in the studies eligible for inclusion in this review. DNAm sites were deemed statistically significant according to the criteria defined in the individual studies. Extracted DNAm sites and corresponding genes were examined to identify any concordance between searches. Mechanistic reviews commonly use tools such as Albatross Plots to quantify the strength of the associations found; however, this was not feasible for this review due to heterogeneity of included studies. Therefore, a narrative synthesis was undertaken [47]. The results and evidence synthesis for this review were reported according to Synthesis Without Meta-analysis guidelines [46,118].

## Supplementary Material

dvag009_Supplemental_Files

## Data Availability

This review uses data from previously published research. All data used can be found in the studies referenced in this review.
