# Supplementary material for: A systematic review investigating the relationship between green and blue spaces and depression in older adults via DNA methylation
Source: Environ Epigenet. 2026 Mar 14;12(1):dvag009. doi: 10.1093/eep/dvag009 (PMC13139854; doi:10.1093/eep/dvag009)
Supplement: dvag009_Supplemental_Files [file dvag009_supplemental_files.zip › Supplementary File 1 Search Strategy.docx]

| Database | Environmental Search Terms | Mental Health Search Terms |
| --- | --- | --- |
| PsycINFO and Embase via Ovid | See Below | See Below |
| Pubmed | **(((((((((((((((((((((((Green Space*) OR (Greenery)) OR (greenness)) OR (Blue space*)) OR (park*)) OR (Parks, Recreational [MeSH])) OR (Tree*)) OR (Forests [MeSH])) OR (Forest*)) OR (Oceans and Seas [MeSH])) OR (Ocean*)) OR (Sea)) OR (NDVI)) OR (NDWI)) OR (Vegetation)) OR (Nature)) OR (coast*)) OR (river*)) OR (lake*)) OR (public space*)) OR (open space*)) OR (garden*)) OR (greenway*)) AND (((((((DNA Methylation [MeSH]) OR (DNA methylation)) OR (hypomethylation)) OR (hypermethylation)) OR (CpG)) OR (Epigenomic*)) OR (epigenetic*)) Filters: Humans, English, from 1000/1/1 - 2024/12/01** | **(((((((Mental health [MeSH]) OR (mental health)) OR (anxiety [MeSH])) OR (anxiety)) OR (depression [MeSH])) OR (Depress*)) OR (Major Depressive Disorder [MeSH])) AND (((((((DNA Methylation [MeSH]) OR (DNA methylation)) OR (hypomethylation)) OR (hypermethylation)) OR (CpG)) OR (Epigenomic*)) OR (epigenetic*)) Filters: Humans, English, from 1000/1/1 - 2024/12/01** |
| MedRxiv and BioRxiv | "green space OR blue space AND epigenetic OR DNA methylation" and posted between "01 December, 2023 and 01 December, 2024" | "mental health OR anxiety OR depression AND epigenetic OR DNA methylation" and posted between "01 December, 2023 and 01 December, 2024" |

**Sample Search Strategy for PsycINFO and Embase via Ovid:**

**GBS and DNA methylation**

| **Set** | **Search Statement** |
| --- | --- |
| **1** | **Green Space*** |
| **2** | **Greenery** |
| **3** | **Greenness** |
| **4** | **Blue Space*** |
| **5** | **Park*** |
| **6** | **Tree*** |
| **7** | **Forest*** |
| **8** | **Ocean*** |
| **9** | **Sea*** |
| **10** | **NDVI** |
| **11** | **NDWI** |
| **12** | **Vegetation** |
| **13** | **Nature** |
| **14** | **Coast*** |
| **15** | **River*** |
| **16** | **Lake*** |
| **17** | **Public space*** |
| **18** | **Open Space*** |
| **19** | **Garden*** |
| **20** | **Greenway*** |
| **21** | **1 or 2 or 3 or 4 or 5 or 6 or 6 or 7 or 8 or 9 or 10 or 11 or 12 or 13 or 14 or 15 or 16 or 17 or 18 or 19 or 20** |
| **22** | **DNA Methylation** |
| **23** | **Hypomethylation** |
| **24** | **Hypermethylation** |
| **25** | **CpG** |
| **26** | **Epigenomic*** |
| **27** | **Epigenetic*** |
| **28** | **22 or 23 or 24 or 25 or 26 or 27** |
| **29** | **21 and 28** |
| **30** | **Limit 29 to English language** |
| **31** | **Limit 30 to human** |
| **32** | **Limit 31 to yr= “1860 –2024”** |
| **Set** | **Search Statement** |
| **1** | **Mental health** |
| **2** | **Anxiety** |
| **3** | **Depress*** |
| **4** | **Depression** |
| **5** | **Major Depressive Disorder** |
| **6** | **1 or 2 or 3 or 4 or 5** |
| **7** | **DNA Methylation** |
| **8** | **Hypomethylation** |
| **9** | **Hypermethylation** |
| **10** | **CpG** |
| **11** | **Epigenomic*** |
| **12** | **Epigenetic*** |
| **13** | **7 or 8 or 9 or 10 or 11 or 12** |
| **14** | **6 and 13** |
| **15** | **Limit 14 to English language** |
| **16** | **Limit 15 to human** |
| **17** | **Limit 16 to yr= “1860 –2024”** |
