# Supplementary material for: A systematic review investigating the relationship between green and blue spaces and depression in older adults via DNA methylation
Source: Environ Epigenet. 2026 Mar 14;12(1):dvag009. doi: 10.1093/eep/dvag009 (PMC13139854; doi:10.1093/eep/dvag009)
Supplement: dvag009_Supplemental_Files [file dvag009_supplemental_files.zip › Supplementary File 2 Inclusion Criteria.docx]

**Search Specific Inclusion Criteria:**

Studies were considered for inclusion if they used a validated outcome measure to investigate participants levels of depression and investigated the relationship between the outcome measure and DNA methylation patterns. This provides a focus at the general population level, compared to studies focusing on clinical populations alone. Further, measurements capture the mental state of participants at the time DNA samples are taken, and can offer insight into symptom severity, which may correlate with DNA methylation changes (44).

A validated, reliable mental health outcome measure must be used for inclusion in this review. The volume of outcome measures has been identified as a problem in mental health research due to the lack of standardisation, leading to the recent development of the Common Measures in Mental Health Science (SMMHS) Initiative (45). This initiative proposed the following outcome measure for research on depression in adults:

Patient Health Questionnaire (PHQ-9)

However, as the CMMHS initiative was established recently (June 2023), studies using any depression outcome measures validated in published research were eligible for inclusion in this review.

**Search Specific Exclusion criteria:**

Due to the dynamic nature of mental health (52,53), longitudinal studies where samples for DNA methylation analysis were collected at a separate stage to psychometric assessment were excluded from this search. If this criterion is not met, it is not possible to capture and associate a participant’s mental state to the DNA methylation changes observed, especially given the reversible nature of DNA methylation (54). Current research also shows there may be correlations between the severity of mental ill health and DNA methylation (44,55), which may be explored if an outcome measure is taken at the time a sample is taken.

There is high comorbidity between neurodevelopmental and neurodegenerative disorders, and mental health difficulties (56–58). Studies focusing primarily on neurodevelopmental conditions, such as autism, or neurodegenerative conditions, such as Alzheimer’s Disease were excluded from the review as existing literature shows these conditions have associated DNA methylation pattern changes (59) that may confound our primary analysis.

DNA methylation is currently being explored in research as a biomarker to predict drug response in patients with mental health conditions (60). Studies that meet the criteria outlined above but associate the DNA methylation changes identified to drug response only, will be excluded. To be eligible for this review, the study must associate the DNA methylation change observed with a psychometric assessment.
