# Supplementary material for: A systematic review investigating the relationship between green and blue spaces and depression in older adults via DNA methylation
Source: Environ Epigenet. 2026 Mar 14;12(1):dvag009. doi: 10.1093/eep/dvag009 (PMC13139854; doi:10.1093/eep/dvag009)
Supplement: dvag009_Supplemental_Files [file dvag009_supplemental_files.zip › Supplementary File 3 Risk of Bias.docx]

**Cross Sectional Studies – BIOCROSS**

| **Lead Author** | **DOI** | **Hypothesis/Objective** | **Study population - selection** | **Study population - representativeness** | **Study population characteristics** | **Statistical analysis** | **Interpretation and evaluation of results** | **Study limitations** | **Specimen characteristics and assay methods** | **Lab measurements** | **Biomarker data modelling** | **Total score** |
| --- | --- | --- | --- | --- | --- | --- | --- | --- | --- | --- | --- | --- |
| **Starnawska** | https://doi.org/10.1038/s41398-019-0548-9 | 2 | 1 | 1 | 1 | 2 | 2 | 0 | 2 | 1 | 1 | 13 |
| **Januar** | https://doi.org/10.1038/tp.2015.114 | 1 | 1 | 1 | 2 | 2 | 2 | 1 | 1 | 1 | 1 | 13 |
| **Ward-Caviness** | https://doi.org/10.1186/s13148-020-00830-8 | 1 | 1 | 1 | 1 | 2 | 2 | 0 | 1 | 1 | 0 | 10 |

Table 1: BIOCROSS scores for studies included in this review.

**BIOCROSS Guidelines**

BIOCROSS Evaluation tool – mentioning all feature of a domain (score of 2), mention of at least 1-2 (score of 1), mention of none (score of 0)

**1st Domain: Study rationale**

1. - Hypothesis/Objective

1.1 Was the biomarker under study described?

1.2 Was the rationale for the study (research question) clearly presented?

1.3 Were the study objectives/ hypothesis clearly stated

**2nd Domain: Design/Methods**

2. - Study population selection

2.1 Were the characteristics of the study participants presented?

2.2 Were the disease stages or comorbidities of the included participants described?

2.3 Were the inclusion and exclusion criteria for study participation defined?

3. - Study population representativeness

3.1 Was the sampling frame reported (study population source)

3.2 Was the participation rate reported (i.e. eligible persons at least 50%)?

3.3 Was sample size justification or power description provided?

**3rd Domain: Data analysis**

4.- Study population characteristics

4.1 Were the study population characteristics (i.e. demographic, clinical and social) presented?

4.2 Were the exposures and potential confounders described?

4.3 Were any missing values and strategies to deal with missing data reported?

5. - Statistical analysis

5.1 Did the authors clearly report statistical methods used to calculate estimates (e.g. Spearman/Pearson/Linear regression, etc.)?

5.2 Were key potential confounding variables measured and adjusted statistically in reported analyses?

5.3 Was the raw effect size estimate (correlation coefficient, beta coefficient) or measure of study precision provided (e.g. confidence intervals, precise (!) p-value*)?,

**4th Domain: Data interpretation**

6. - Interpretation and evaluation of results

6.1 Was the data discussed in the context of study objectives/hypotheses?

6.2 Was the interpretation of the results considering findings from similar studies?

6.3 Was the biological context described?

7. Study limitations

7.1 Was the cross-sectional nature of the analysis discussed?

7.2 Did the authors acknowledge restricted interpretation due to measurements at one point in time and no statement about causality possible using cross-sectional studies?

7.3 Did the authors acknowledge need for consistency with other research?

**5th Domain: Biomarker measurement**

8. Specimen characteristics and assay methods

8.1 Were the measurement methods described? (assay methods, preservation and storage, detailed protocol, including specific reagents or kits used)

8.2 Were the reproducibility assessments performed for evaluating biomarker stability?

8.3 Were the quantitation methods well described?

9. Laboratory measurement

9.1 Was the laboratory/place of measurement mentioned?

9.2 Were any quality control procedures and results reported (e.g. reported coefficient of variation?

9.3 Were the analyses blinded for laboratory staff?

10. Biomarker data modelling

10.1 Was the distribution of biomarker data reported (if non-normal how it was standardized)?

10.2 Did the authors report on methods or outlier detection and handling?

10.3 Were any possible errors resulting from measurement inaccuracies discussed?

*Reporting not significant (ns) or p > 0.05 is not precise and does not allow a judgment on precision

**Cohort Studies – Joanna Briggs**

| **Lead Author** | **DOI** | **Population** | **Exposures** | **Exposure measures** | **Confounding factors identified** | **Confounding factors mitigated** | **Participants free of outcome prior to exposure** | **Valid and reliable outcome measures** | **Follow up - time** | **Follow up completed?** | **Strategies to address incomplete follow up** | **Statistical analysis** | **Score** |
| --- | --- | --- | --- | --- | --- | --- | --- | --- | --- | --- | --- | --- | --- |
| **Xu** | **https://doi.org/10.1016/j.envint.2021.106556** | **Y** | **Y** | **Y** | **Y** | **Y** | **N** | **Y** | **N** | **NA** | **NA** | **Y** | **7** |
| **Jeong** | **https://doi.org/10.1016/j.envint.2021.106945** | **Y** | **Y** | **Y** | **Y** | **UC** | **N** | **Y** | **Y** | **Y** | **NA** | **Y** | **8** |
| **Vos** | **https://doi.org/10.1016/j.psyneuen.2024.107088** | **Y** | **Y** | **Y** | **Y** | **Y** | **N** | **Y** | **Y** | **N** | **N** | **Y** | **8** |
| **Kang (2015)** | **https://doi.org/10.1016/j.neurobiolaging.2014.12.035** | **Y** | **Y** | **Y** | **Y** | **Y** | **N** | **Y** | **Y** | **U** | **U** | **Y** | **8** |
| **Kang (2018)** | https://doi.org/10.1016/j.pnpbp.2018.02.004 | **Y** | **Y** | **Y** | **Y** | **Y** | **N** | **Y** | **Y** | **Y** | **Y** | **Y** | **10** |
| **Liao** | **https://doi.org/10.1016/j.gerinurse.2022.01.015** | **Y** | **Y** | **Y** | **N** | **N** | **N** | **Y** | **U** | **Y** | **NA** | **Y** | **6** |

Table 2: Joanna Briggs cohort study assessment tool scores

**Guidance for Joanna Briggs:**

JBI Critical Appraisal Checklist for cohort studies

Reviewer ______________________________________ Date_______________________________

Author_______________________________________ Year_________ Record Number_________

|  | Yes | No | Unclear | Not applicable |
| --- | --- | --- | --- | --- |
| 1. Were the two groups similar and recruited from the same population? | □ | □ | □ | □ |
| 1. Were the exposures measured similarly to assign people to both exposed and unexposed groups? | □ | □ | □ | □ |
| 1. Was the exposure measured in a valid and reliable way? | □ | □ | □ | □ |
| 1. Were confounding factors identified? | □ | □ | □ | □ |
| 1. Were strategies to deal with confounding factors stated? | □ | □ | □ | □ |
| 1. Were the groups/participants free of the outcome at the start of the study (or at the moment of exposure)? | □ | □ | □ | □ |
| 1. Were the outcomes measured in a valid and reliable way? | □ | □ | □ | □ |
| 1. Was the follow up time reported and sufficient to be long enough for outcomes to occur? | □ | □ | □ | □ |
| 1. Was follow up complete, and if not, were the reasons to loss to follow up described and explored? | □ | □ | □ | □ |
| 1. Were strategies to address incomplete follow up utilized? | □ | □ | □ | □ |
| 1. Was appropriate statistical analysis used? | □ | □ | □ | □ |

Overall appraisal: Include □ Exclude □ Seek further info □

Comments (Including reason for exclusion)

________________________________________________________________________________________________________________________________________________________________________________________________

Explanation of cohort studies critical appraisal

How to Cite: *Moola S, Munn Z, Tufanaru C, Aromataris E, Sears K, Sfetcu R, Currie M, Qureshi R, Mattis P, Lisy K, Mu P-F. Chapter 7: Systematic reviews of etiology and risk . In: Aromataris E, Munn Z (Editors)*. JBI Manual for Evidence Synthesis. JBI, 2020. Available from <https://synthesismanual.jbi.global>

## Cohort Studies Critical Appraisal Tool

Answers: Yes, No, Unclear or Not/Applicable

## 1. Were the two groups similar and recruited from the same population?

Check the paper carefully for descriptions of participants to determine if patients within and across groups have similar characteristics in relation to exposure (e.g. risk factor under investigation). The two groups selected for comparison should be as similar as possible in all characteristics except for their exposure status, relevant to the study in question. The authors should provide clear inclusion and exclusion criteria that they developed prior to recruitment of the study participants.

## 2. Were the exposures measured similarly to assign people to both exposed and unexposed groups?

A high quality study at the level of cohort design should mention or describe how the exposures were measured. The exposure measures should be clearly defined and described in detail. This will enable reviewers to assess whether or not the participants received the exposure of interest.

## 3. Was the exposure measured in a valid and reliable way?

The study should clearly describe the method of measurement of exposure. Assessing validity requires that a 'gold standard' is available to which the measure can be compared. The validity of exposure measurement usually relates to whether a current measure is appropriate or whether a measure of past exposure is needed.

Reliability refers to the processes included in an epidemiological study to check repeatability of measurements of the exposures. These usually include intra-observer reliability and inter-observer reliability.

## 4. Were confounding factors identified?

Confounding has occurred where the estimated intervention exposure effect is biased by the presence of some difference between the comparison groups (apart from the exposure investigated/of interest). Typical confounders include baseline characteristics, prognostic factors, or concomitant exposures (e.g. smoking). A confounder is a difference between the comparison groups and it influences the direction of the study results. A high quality study at the level of cohort design will identify the potential confounders and measure them (where possible). This is difficult for studies where behavioral, attitudinal or lifestyle factors may impact on the results.

## 5. Were strategies to deal with confounding factors stated?

Strategies to deal with effects of confounding factors may be dealt within the study design or in data analysis. By matching or stratifying sampling of participants, effects of confounding factors can be adjusted for. When dealing with adjustment in data analysis, assess the statistics used in the study. Most will be some form of multivariate regression analysis to account for the confounding factors measured. Look out for a description of statistical methods as regression methods such as logistic regression are usually employed to deal with confounding factors/variables of interest.

## 6. Were the groups/participants free of the outcome at the start of the study (or at the moment of exposure)?

The participants should be free of the outcomes of interest at the start of the study. Refer to the ‘methods’ section in the paper for this information, which is usually found in descriptions of participant/sample recruitment, definitions of variables, and/or inclusion/exclusion criteria.

## 7. Were the outcomes measured in a valid and reliable way?

Read the methods section of the paper. If for e.g. lung cancer is assessed based on existing definitions or diagnostic criteria, then the answer to this question is likely to be yes. If lung cancer is assessed using observer reported, or self-reported scales, the risk of over- or under-reporting is increased, and objectivity is compromised. Importantly, determine if the measurement tools used were validated instruments as this has a significant impact on outcome assessment validity.

Having established the objectivity of the outcome measurement (e.g. lung cancer) instrument, it’s important to establish how the measurement was conducted. Were those involved in collecting data trained or educated in the use of the instrument/s? (e.g. radiographers). If there was more than one data collector, were they similar in terms of level of education, clinical or research experience, or level of responsibility in the piece of research being appraised?

## 8. Was the follow up time reported and sufficient to be long enough for outcomes to occur?

The appropriate length of time for follow up will vary with the nature and characteristics of the population of interest and/or the intervention, disease or exposure. To estimate an appropriate duration of follow up, read across multiple papers and take note of the range for duration of follow up. The opinions of experts in clinical practice or clinical research may also assist in determining an appropriate duration of follow up. For example, a longer timeframe may be needed to examine the association between occupational exposure to asbestos and the risk of lung cancer. It is important, particularly in cohort studies that follow up is long enough to enable the outcomes. However, it should be remembered that the research question and outcomes being examined would probably dictate the follow up time.

## 9. Was follow up complete, and if not, were the reasons to loss to follow up described and explored?

It is important in a cohort study that a greater percentage of people are followed up. As a general guideline, at least 80% of patients should be followed up. Generally a dropout rate of 5% or less is considered insignificant. A rate of 20% or greater is considered to significantly impact on the validity of the study. However, in observational studies conducted over a lengthy period of time a higher dropout rate is to be expected. A decision on whether to include or exclude a study because of a high dropout rate is a matter of judgement based on the reasons why people dropped out, and whether dropout rates were comparable in the exposed and unexposed groups.

Reporting of efforts to follow up participants that dropped out may be regarded as an indicator of a well conducted study. Look for clear and justifiable description of why people were left out, excluded, dropped out etc. If there is no clear description or a statement in this regards, this will be a 'No'.

## 10. Were strategies to address incomplete follow up utilized?

Some people may withdraw due to change in employment or some may die; however, it is important that their outcomes are assessed. Selection bias may occur as a result of incomplete follow up. Therefore, participants with unequal follow up periods must be taken into account in the analysis, which should be adjusted to allow for differences in length of follow up periods. This is usually done by calculating rates which use person-years at risk, i.e. considering time in the denominator.

## 11. Was appropriate statistical analysis used?

As with any consideration of statistical analysis, consideration should be given to whether there was a more appropriate alternate statistical method that could have been used. The methods section of cohort studies should be detailed enough for reviewers to identify which analytical techniques were used (in particular, regression or stratification) and how specific confounders were measured.

For studies utilizing regression analysis, it is useful to identify if the study identified which variables were included and how they related to the outcome. If stratification was the analytical approach used, were the strata of analysis defined by the specified variables? Additionally, it is also important to assess the appropriateness of the analytical strategy in terms of the assumptions associated with the approach as differing methods of analysis are based on differing assumptions about the data and how it will respond.
